# Use of Fourier-Transform Infrared Spectroscopy (FT-IR) for Monitoring Experimental *Helicobacter pylori* Infection and Related Inflammatory Response in Guinea Pig Model

**DOI:** 10.3390/ijms22010281

**Published:** 2020-12-30

**Authors:** Weronika Gonciarz, Łukasz Lechowicz, Mariusz Urbaniak, Wiesław Kaca, Magdalena Chmiela

**Affiliations:** 1 Department of Immunology and Infectious Biology, Institute of Microbiology, Biotechnology and Immunology, Faculty of Biology and Environmental Protection, University of Lodz, Banacha 12/16, 90-237 Łódź, Poland; weronika.gonciarz@biol.uni.lodz.pl; 2 Institute of Biology, Faculty of Natural Sciences, Jan Kochanowski University, Uniwersytecka 7, 25-406 Kielce, Poland; lukasz.lechowicz@ujk.edu.pl (Ł.L.); wieslaw.kaca@ujk.edu.pl (W.K.); 3 Institute of Chemistry, Faculty of Natural Sciences, Jan Kochanowski University, Uniwersytecka 7, 25-406 Kielce, Poland; mariusz.urbaniak@ujk.edu.pl

**Keywords:** *H. pylori*, FT-IR, guinea pigs, chemometric, cluster analysis

## Abstract

Infections due to Gram-negative bacteria *Helicobacter pylori* may result in humans having gastritis, gastric or duodenal ulcer, and even gastric cancer. Investigation of quantitative changes of soluble biomarkers, correlating with *H. pylori* infection, is a promising tool for monitoring the course of infection and inflammatory response. The aim of this study was to determine, using an experimental model of *H. pylori* infection in guinea pigs, the specific characteristics of infrared spectra (IR) of sera from *H. pylori* infected (40) vs. uninfected (20) guinea pigs. The *H. pylori* status was confirmed by histological, molecular, and serological examination. The IR spectra were measured using a Fourier-transform (FT)-IR spectrometer Spectrum 400 (PerkinElmer) within the range of wavenumbers 3000–750 cm^−1^ and converted to first derivative spectra. Ten wavenumbers correlated with *H. pylori* infection, based on the chi-square test, were selected for a K-nearest neighbors (k-NN) algorithm. The wavenumbers correlating with infection were identified in the W2 and W3 windows associated mainly with proteins and in the W4 window related to nucleic acids and hydrocarbons. The k-NN for detection of *H. pylori* infection has been developed based on chemometric data. Using this model, animals were classified as infected with *H. pylori* with 100% specificity and 97% sensitivity. To summarize, the IR spectroscopy and k-NN algorithm are useful for monitoring experimental *H. pylori* infection and related inflammatory response in guinea pig model and may be considered for application in humans.

## 1. Introduction

*Helicobacter pylori* (*H. pylori*) is a Gram-negative, microaerophilic, pathogenic bacterium, colonizing the human gastric mucosa. These bacteria were isolated for the first time by Marshall and Warren in 1984 from the stomachs of patients with gastritis [1]. About 50% of the world population is infected with *H. pylori.* However, there are areas where the rate of infection reaches 80–90% [2]. The infection occurs most often in childhood and if left untreated, can persist throughout life. *H. pylori* is an etiological agent of chronic gastritis, gastric and duodenal ulcers, malignant diseases: mucosa-associated lymphoid tissue lymphoma (MALT), and gastric cancer [2,3,4,5,6,7]. *H. pylori* induce cellular and humoral immune responses of the host. However, the chronic character of *H. pylori* infections suggests that the immune system is not able to eradicate these bacteria [8,9].

Some *H. pylori* antigens, including urease and vacuolating cytotoxic (VacA) or cytotoxin associated gene A (CagA) protein increase the inflammatory response, while others such as lipopolysaccharide (LPS) inhibit the activity of immune cells [10,11,12,13]. Knowledge about the pathogenesis and different courses of *H. pylori* infections is insufficient. Therefore, animal models that follow the natural history of *H. pylori* infection and related inflammatory and immune processes are needed. So far, mice, Mongolian gerbils, guinea pigs (*Caviae porcellus*), dogs, cats, pigs, and monkeys have been used [14,15]. Guinea pigs are considered the optimal model because they share similarities with humans in terms of immune responses, complement proteins, thymus and bone marrow physiology, pulmonary physiology, and corticosteroid response [16,17,18]. Guinea pigs are not naturally infected with *Helicobacter* spp. However, they are susceptible to *H. pylori* infection, which can be confirmed by histological examination of gastric tissue specimens for inflammatory response and *Helicobacter*-like organisms, detection of specific nucleotide sequences using molecular methods such as polymerase chain reaction (PCR), and serological examination of serum samples for specific anti-*H. pylori* antibodies or *H. pylori* antigens in stool samples [19].

The varied course of *H. pylori* infections prompts researchers to search for new diagnostic methods that would enable the determination of soluble markers qualitatively and quantitatively, which would be helpful in understanding the course of infection and its consequences. Recently, fast physical methods like Fourier-transform infrared spectroscopy (FT-IR) began to be used for the diagnosis of diseases and for monitoring cellular alterations based on spectral analysis of biological fluids such as blood, serum, saliva, and urine [20,21].

There are two main types of IR spectroscopy: transmission spectroscopy, in which the intensity of radiation passing through the sample is measured and reflection spectroscopy, in which the intensity of the IR radiation reflected by the tested sample is measured. In the case of reflection spectroscopy, there are three techniques of measurement: attenuated total reflectance (ATR), diffuse reflectance infrared Fourier transform spectroscopy (DRIFTS), and FT-IR microscopy. In the ATR technique, the IR radiation passes through the crystal and the tested sample closely adheres to its surface. As a result of the total reflection of the IR beam from the crystal surface, an evanescent wave is created which penetrates the sample. The examples of the use of ATR-IR in biological samples analysis are summarized in Table 1.

FT-IR spectra of biological samples can be divided into groups of components with typical absorption bands in the wavenumber windows (W):W1—fatty acids (wavenumber range of 3000 cm^−1^ to 2800 cm^−1^), W2—peptides and proteins (wavenumber range of 1800 cm^−1^ to 1500 cm^−1^), W3—proteins, phosphate-carrying compounds and fatty acids (wavenumber range of 1500 cm^−1^ to 1200 cm^−1^), W4—carbohydrates (wavenumber range of 1200 cm^−1^ to 900 cm^−1^). The window W5 (wavenumber range of 900 cm^−1^ to 750 cm^−1^) corresponds to specific peaks unique for the biological samples [52,53,54,55]. The aim of this study was to determine the specific characteristics of IR spectra for *H. pylori* infected guinea pigs in terms of production of antibodies as well as inflammatory and metabolic biomarkers related to experimentally induced infection. The potential usefulness of this technique in the guinea pig model presumably will allow the application of this methodology to the analysis of human biological samples.

## 2. Results

### 2.1. H. pylori Status

In our model, the status of *H*. *pylori* infection in guinea pigs was confirmed at 28 days after inoculation of animals with these bacteria, by histological, molecular, and serological methods, as previously described [56,57]. The gastric mucosa of guinea pigs inoculated with *H*. *pylori* was colonized by bacteria, as shown by Giemsa and Warthin-Starry staining to detect *Helicobacter*-like organisms. Tissue staining with hematoxylin and eosin also showed inflammation within the gastric mucosa. In the gastric tissue of infected animals, *cagA* and *ureC* sequences encoding CagA protein and subunit C of urease, respectively, were detected by polymerase chain reaction. These sequences were not detected in noninfected animals. The infected animals responded to *H. pylori* bacteria by producing anti-*H*. *pylori* immunoglobulins (Igs) of IgM and IgG class (Table 1). The level of serum IgM and IgG antibodies towards *H. pylori* antigens, which were detected with use of glycine acid extract (GE) from the reference strain, was significantly higher in *H. pylori* infected animals compared to noninfected animals (Table 2).

**Figure 1:**
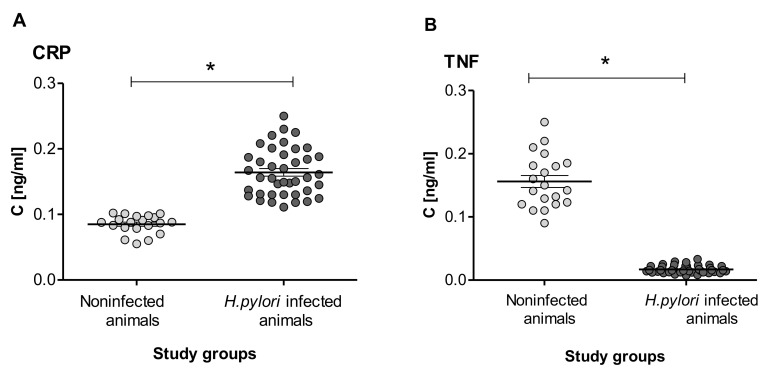
The level of (**A**)—CPR and (**B**)—TNF in guinea pig sera. Samples collected from noninfected guinea pigs (20 per group) and *H. pylori* infected guinea pigs (40 per group) were examined. The results are presented as mean values ± standard deviation (SD), for each experimental variant. Statistical analysis was performed with the nonparametric Mann–Whitney U test. Statistical significance * *p* < 0.05, was shown for *H. pylori* infected vs. control animals. CRP: C-reactive protein. TNF: tumor necrosis factor.

The level of C-reactive protein (CRP), which is a key systemic marker of inflammation, in serum samples from *H. pylori* infected animals, was also upregulated (Figure 1A). The level of tumor necrosis factor (TNF), which is a pro-inflammatory cytokine, in these samples was significantly lower than that in samples from control animals (Figure 1B).

### 2.2. Analysis of IR Spectra of Guinea Pig Sera

**Figure 2:**
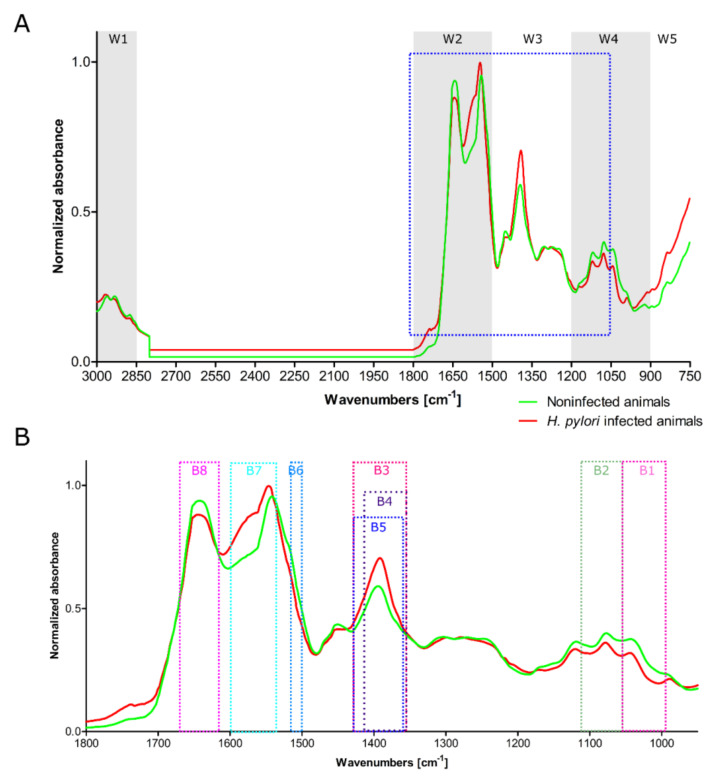
Representative infrared spectra of guinea pig sera. Red—*H. pylori* positive animals. Green—*H. pylori* negative animals. (**A**) The full-range IR spectra. Range of the IR spectrum marked in blue that best differentiated between *H. pylori* infected and noninfected animals. (**B**) Characteristic absorption bands B1–B8 considered in the analysis.

The IR spectra of serum samples can be divided into different windows (W) corresponding to different components characterized by absorption bands: fatty acids (Window—W1: wavenumber range of 3000 cm^−1^ to 2800 cm^−1^); peptides and proteins (W2: wavenumber range of 1800 cm^−1^ to 1500 cm^−1^); proteins, phosphate-carrying compounds and fatty acid (W3: wavenumber range of 1500 cm^−1^ to 1200 cm^−1^); W4—carbohydrates (wavenumber range of 1200 cm^−1^ to 900 cm^−1^). The window W5 (wavenumber range of 900 cm^−1^ to 750 cm^−1^) corresponds to a specific peak unique to biological samples (Figure 2).

Vibration band assignment was carried out in the infrared spectra of sera by comparing the position, relative intensity, and shape of the bands with the corresponding features of the associated molecular bands. The infrared spectra of serum samples from uninfected and *H. pylori* infected animals are shown in Figure 2. The absorption bands of proteins, lipids, and carbohydrates were considered during the analysis.

Specific molecules, which were identified in the composition of the IR spectra of guinea pig sera, such as glucose (part B1), α2-globulins (part B2), IgM (part B3), IgG1 (part B4), transferrin (part B5), IgG4 (part B6), CRP (part B7), and TNF (part B8), allowed the differentiation between *H. pylori* infected and uninfected animals (Figure 2A,B and Table 3). A vibration band assignment was made concerning frequencies of the chemical groups present in the sample. As shown in Table 2, in the spectral region (B1) corresponding to glucose, the prominent absorption band at 1062–997 cm^−1^ is due to the C–O symmetric stretching of the glucose region and the C–O stretching in carboxylic acids. The absorption band B2 (component group: amino acid), is characterized by the α2-globulins absorption vibration peaks at: 1060–116 cm^−1^ C–C–C bending, C–O stretching, and C–N stretching (Figure 2 and Table 3). The spectral region B3, which is typical of proteins, phosphate-carrying compounds, and fatty acids (component group of carboxylic acid and amino acid), corresponds to the vibration band characteristics of IgM antibodies. The typical parameters of this region are as follows: N–O symmetric stretching, O–H bending, and methyl symmetric deformation. The main absorption peak identified for IgG was 1419–1361cm^−1^ (B4), due to C–H rocking, C–C stretching, and methyl symmetric deformations. The absorption peaks of transferrin were identified at 1428–1363 cm^−1^ (B5), and can include chemical bonds: CH_2_ wagging and O–H bending of carboxylic acid. The absorption bands of IgG4 (B6) at 1538–1505 cm^−1^ correspond to C–N stretching and NH bending in amide II. The spectral region between 1541 cm^−1^ and 1600 cm^−1^ (B7, CRP) is predominantly related to C–C stretching, NH bending, and N–H in plane bending vibrations strongly coupled with C–N stretching of protein (amide II). The C=O symmetric stretching, C=C stretching, and NH_2_ scissoring vibrations were found at 1652–1628 cm^−1^ (B8, TNF) (Figure 2A,B and Table 1).

### 2.3. Wavenumber Correlating with H. pylori Infection and Mathematical Models Identifying Sera of Infected Individuals

The regions of the infrared spectra that are correlated with *H. pylori* infection in guinea pigs were determined by using the chi-square statistical test. To perform the cluster analysis (HCA), 10 wavenumbers strongly correlated with this infection were considered (Table 4).

**Figure 3:**
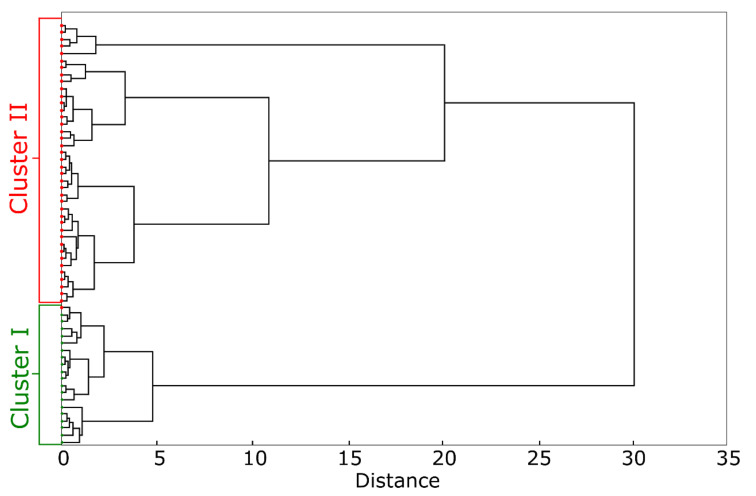
The cluster analysis based on the first derivative of IR spectra of guinea pig sera. Upper branches on the dendrogram (Cluster II)—*H. pylori* infected animals. Lower branches on the dendrogram (Cluster I)—noninfected animals. The dendrogram was calculated using Ward‘s method and Manhattan length.

The HCA analysis of the W2–W4 regions of IR spectra and their first derivatives showed two distinct clusters (Figure 3). Cluster I corresponds to noninfected animals, while cluster II is representative for *H. pylori* infected animals. One wavenumber appears in the W4 window, which is associated with the vibrations generated by hydrocarbons and nucleic acids. The next five wavenumbers were correlated with *H. pylori* infection in the window W3. This window is associated with vibrations of different bonds (so-called mixed region); however, proteins and phosphorus-containing compounds are key to this window.

The wavenumbers of 1552 cm^−1^, 1541 cm^−1^, and 1630 cm^−1^, localized within the W2 window were strongly associated with *H. pylori* infection. According to the literature this window is characteristic for proteins and peptides. The peaks in this region of the spectrum for *H. pylori* infected animals may reflect production of antibodies and/or an increased production of inflammatory proteins. In *H. pylori* infected animals, it may correspond to the production of antibodies and/or greater production of inflammatory proteins. The entire IR spectra range (W2–W4) has been used to develop a k-nearest neighbor predictive model to discriminate *H. pylori* infected from uninfected animals. Our model is characterized by a sensitivity of 97% and a specificity of 100% (Table 5).

Regardless of this model, we also showed that all the wavenumbers considered in the analysis IR spectra of the analyzed sera included in Table 3 were also useful for differentiation of serum samples. The average values of χ2-square test or *p*-value for the wavenumbers differ significantly (*p*  <  0.0001, *t*-test) between *H. pylori* infected and noninfected animas. According to the Shapiro–Wilk test, the distribution of the wavenumber values in both groups was normal (*p*  =  0.59 for *H. pylori* infected animals and *p*  =  0.22 for uninfected animals).

*H. pylori* infected animals were distinguished from the control group by the following rule: if the value of the first-order derivative of all wavenumbers in the IR spectra of *H. pylori* infected animals vs. uninfected animals, included in Table 4, is <0.01, then the animals will be treated as *H. pylori* infected.

## 3. Discussion

The gold standard for detection of *H. pylori* infection in humans consists of an invasive endoscopic technique, during which gastric tissue samples are taken for further examination by a rapid urease test (RUT), histological staining for the presence of *Helicobacter*-like organisms (HLO) and inflammatory cells and/or culture [62,63]. Among non-invasive methods, the ^13^C urea breath test (UBT) is generally accepted as sufficiently specific and sensitive for primary diagnosis and confirming the effectiveness of eradication therapy [64,65]. Many variants of ELISA test are used for detection of specific anti-*H. pylori* antibodies in serum samples. Detection of specific *H. pylori* DNA sequence in the gastric tissue and *H. pylori* antigens in stool samples was considered as a diagnostic tool [63]. However, a comprehensive method is needed to quantify selected markers to track the systemic effects of the infection that result from *H. pylori* colonization of gastric mucosa. Potentially, the determination of such markers may be helpful in predicting the course of infection and its consequences. It may also contribute to the confirmation of the relationship between local *H. pylori* infection and the development of extragastric diseases, including coronary heart disease, anemia, and type 2 diabetes [66,67].

In our study we used IR spectroscopy, which has been adopted for the characterization of biological materials, like tissue sections, cytological and histological specimens or biofluids, particularly serum samples. Using this technique to analyze serum samples, Ghimire et al. [68] identified mouse lymphoma and melanoma, while Gajjaee et al. [28] showed spectral changes in blood samples from patients with ovarian cancer. IR spectroscopy was useful for the estimation of serum cortisol levels in athletes [69], whereas Lechowicz et al. [29] used FT-IR spectroscopy to enable the differentiation between sera from patients with rheumatoid arthritis (RA) and non-RA sera. Zhou et al. [70] showed spectral changes in the HL60 cell line during various stages of cell differentiation and apoptosis. Erukhimovitch et al. [27] analyzed human plasma by FT-IR to determine spectral parameters for monitoring patients with leukemia. Shen et al. [71] used FT-IR to develop a noninvasive method to measure glucose in whole blood samples from diabetic patients [71], whereas Sankari et al. [40] analyzed serum immunoglobulins (IgA, IgM, and IgG) in patients with myeloma. FT-IR can be potentially useful for monitoring chronic infections based on changes in the systemic concentration of selected exogenous and endogenous molecules. We showed that FT-IR may help to investigate quantitative changes of selected soluble biomarkers, correlated with *H. pylori* infection in children and presumable consequent delayed growth [35].

Previously we developed an experimental model of *H. pylori* infection in guinea pigs, which was followed by the inflammatory response in gastric tissue, similarly to humans infected with these bacteria [56,57,72]. This model allowed us to study the outcome of *H*. *pylori*-driven inflammatory and immune responses as well as induction of regeneration processes in the gastric tissue in response to this infection [72,73]. The effects of *H. pylori* infections can vary from gastritis to gastric ulcer, duodenal ulcer, and gastric cancer, but the reasons for this are unknown. Therefore, new diagnostic methods need to be developed to assess *H. pylori* infection and its potential consequences. Such possibilities are offered by the experimental model of *H. pylori* infection in guinea pigs.

The aim of this study was to combine HCA analysis to determine the fragments of IR spectra of guinea pig sera characteristic for *H. pylori* infection. For this purpose, we used sera of uninfected animals (*n* = 20) or animals experimentally infected with *H. pylori* (*n* = 40). *H. pylori* infection was confirmed in all guinea pigs that received *H. pylori,* as previously described [56,57,73]. Infrared spectra were measured using an FT-IR Spectrum 400 spectrometer in the range of wavenumber 3000–750 cm^−1^ and then subjected to mathematical pre-processing (calculation of first derivative).

Sera from *H. pylori* infected animals showed lower absorbance values for TNF, α2-globulins, and glucose, while higher absorbance values were detected for CRP, transferrin, as well as IgM, IgG1/IgG4 immunoglobulins, than in sera from *H. pylori* uninfected animals.

The CRP protein is a widely accepted systemic marker of the inflammatory response, in *H. pylori* infection [74,75]. We showed elevated levels of CRP in serum samples of *H. pylori* infected compared to uninfected animals, which was consistent with the development of an inflammatory response in the gastric tissue colonized with these bacteria. By comparison, levels of TNF in serum from *H. pylori* infected animals were significantly lower than in uninfected animals, which remains in line with a lower maximum absorption of the characteristic wavenumber of this cytokine.

This remains in line with a lower maximum absorption of the characteristic wavenumber for this cytokine. It is worth mentioning that in humans, *H. pylori* status was found to correlate with serum levels of CRP. In turn, increased levels of CRP were associated with elevated levels of total cholesterol and triglycerides, which are the risk factors for CHD [76]. The production of CRP in the liver is regulated by proinflammatory TNF. However, in this study we showed using FT-IR that TNF was lower in serum samples of *H. pylori* infected animals than uninfected animals. It could be due to neutralization of this cytokine by soluble TNF receptor. As shown by Shibata et al., during *H. pylori* infection the gastric epithelial cells undergo TNF-driven apoptosis and due to this the host responds by elevated production of soluble TNF receptor I in order to neutralize TNF and prevent its deleterious effects [77]. It is possible that in this study the lower level of TNF in the sera of guinea pigs infected with *H. pylori* as compared to uninfected animals may be due to neutralization of this cytokine by soluble TNF receptor. Previously we showed that *H. pylori* infection in guinea pigs was related to increased oxidative stress and apoptosis, and that the serum level of TNF in *H. pylori* infected animals determined by ELISA was lower than in uninfected animals [56,57].

The absorbance values of glucose and α2-globulins were also lower in *H. pylori* infected animals than in uninfected animals. The relationship between *H. pylori* infection and lower fasting blood glucose level has been previously reported [78,79,80]. Higher absorbance values shown for IgM, IgG1, IgG4, and transferrin when testing sera from *H. pylori* infected guinea pigs are consistent with higher levels of anti-*H. pylori* antibodies in this group, as evaluated by ELISA. In *H. pylori* infected patients, a higher systemic level of transferrin has been observed in conjunction with iron deficiency [81,82].

The study designed a predictive mathematical model: k-NN algorithm to distinguish *H. pylori* infected animals from uninfected animals with a sensitivity of 97% and a specificity of 100%. The proposed model can be a useful tool to detect and track the course of *H. pylori* infection, considering the serological and inflammatory parameters. Only one serum from the group of *H. pylori* infected animals was misclassified based on IR spectra. This could have been caused by the water in the samples which may affect the IR spectrum in the entire spectral range. Therefore, further studies are necessary to develop standardized protocols for the samples used for analysis.

## 4. Materials and Methods

### 4.1. Ethics Statement

In vivo experiments were developed according to the EU directive (Directive 2010/63/EU of the European Parliament and of the Council of 22 September 2010 on the protection of animals used for scientific purposes (Dz.U. L 276 z 20.10.2010, s. 33–79), and were approved by the resolution of the Local Ethics Committee (LKE9) for Animal Experiments of the Medical University of Lodz, Poland, which was established by the Ministry of Science and Higher Education in Poland (Decision 58/ŁB45/2016).

### 4.2. H. pylori Infection in Caviae porcellus (Guinea Pigs)

Three-month-old, male Himalayan guinea pigs (400–600 g), free of pathogens, were housed in the Animal House at the Faculty of Biology and Environmental Protection, University of Lodz (Poland), kept in cages with free access to drinking water and fed with standard chow. The animals were inoculated *per os* with bacterial suspension of the reference *H. pylori* strain CCUG 17874 (Culture Collection University of Gothenburg, Sweden), three times in two day intervals as previously described [56,57]. Then, 28 days after the last *H. pylori* inoculation, the animals were euthanized according to a protocol approved by an ethics committee, and the gastric tissue was collected for analyses, whereas blood samples were processed to obtain serum and then stored at −80 °C. *H. pylori* infection was confirmed by microscopic imaging of HLOs, scoring tissue inflammation in thin layer preparations stained by routine histological staining, polymerase chain reaction (PCR) to detect *H. pylori cagA* and *ureC* gene sequences, as previously described [56].

The laboratory ELISA for anti-*H. pylori* IgM and IgG antibodies against the *H. pylori* antigenic complex, glycine extract (GE), which was obtained by extraction with glycine acid buffer of surface antigens from the reference *H. pylori* 17874 strain CCUG, producing CagA protein and VacA, as previously described [83]. Major proteins in GE recognized by the reference sera from *H. pylori* infected patients were as follows: 120 kDa (CagA), 87 kDa (VacA), 66 kDa (UreB, subunit B of urease), 60 kDa (heat shock protein—Hsp), 29 kDa (UreA), between 66–22 kDa [65]. The GE protein concentration was 600 μg/mL (NanoDrop 2000c Spectrophotometer, ThermoScientific, Wlatman, MA, USA) and <0.001 EU/mL of LPS, as shown by the chromogenic Limulus amebocyte lysate test (Lonza, Braine-Alleud, Belgium) as previously described [83,84].

In total, 60 animals were used in the study: 20 uninfected (control) and 40 infected with *H. pylori*. The levels of anti-*H. pylori* GE IgM and IgG were showed as mean ± standard deviation (SD) for each group.

### 4.3. ELISA for C-Reactive Protein (CRP) and Tumor Necrose Factor (TNF)

Total CPR or TNF concentrations in guinea pig serum samples were determined by ELISA with a sensitivity of 4 pg/mL for CRP (Cloud-Clone, Katy, TX, USA) and 8 pg/mL for TNF (ThermoScientific, Waltham, MA, USA), as recommended by the manufacturer. The absorbance was estimated using a Victor2 reader at a wavenumber of 450 nm. For each group the median values were calculated.

### 4.4. The Measurement of Infrared Spectra and Its Processing

IR spectra of animal sera were evaluated using the ATR-FTIR technique using the FT-IR/FT- near-infrared spectroscopy (NIR) Spectrum 400 spectroscope (Perkin Elmer, Waltham, MA, USA). The spectrometer was equipped with an ATR single reflection diamond crystal. Serum samples were stored at −80 °C until the measurement was made. Sera samples were thawed at 20 °C using a CH-100 (BIOSAN, Riga, Latvia) thermoblock and then shaken for 30 s using LabDancer vario (IKA, Staufen im Breisgau, Germany). Measurements were made at 20 °C with constant air humidity. Before each measurement, the ATR crystal was thoroughly cleaned with 95% alcohol and a baseline measurement was made. One microliter of serum was added to the spectroscope crystal by using a disposable sterile pipette tip and was left to evaporate the water. The amount of evaporated water was estimated based on the intensity of a band at 3400–3200 cm^−1^. It was assumed that the water was evaporated from the sample when the band intensity at wavenumber 3300 cm^−1^ decreased to about 40% of the original value. In such conditions, the process of water evaporation lasted about 5 min. The high water content in the sample suppresses the intensity of the bands throughout the IR spectrum. After removing most of the water from the sample, the spectrum of the sediment remaining on the ATR crystal was measured. IR spectra were measured in the wavenumber range of 4000–650 cm^−1^ with a resolution of 1 cm^−1^ and then preprocessed in two steps: (a) calculation of the first derivative by a five-point stencil, (b) normalization to this range {0.1} [29].

**Figure 4:**
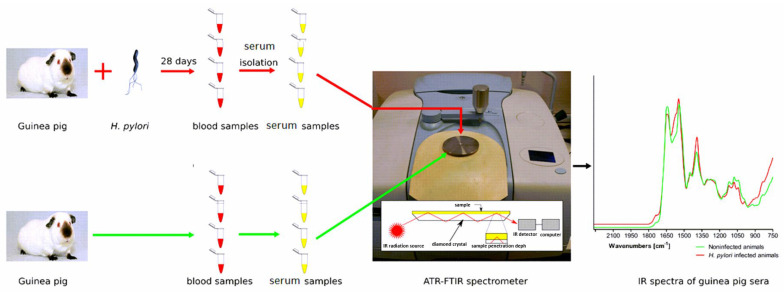
The general scheme of the analysis of guinea pig sera by FT-IR method.

The first derivatives were used for mathematical analysis. To develop the dendrograms, we used the Manhattan metric and Ward’s method. Serum samples were collected from each guinea pig in four test tubes. One IR spectrum for each serum sample was collected as a result of 25 running scans. A total number of 240 IR spectra were measured (Figure 4.)

### 4.5. Mathematical Model Developing for Guinea Pigs Differentiation

For developing a prediction model we used k-NN algorithm. The k-NN classifier is one of the well-known and simple classification algorithms. It was first introduced by Fix and Hodges as a non-parametric algorithm, i.e., it does not make any assumptions on the input data distribution; thus, it is widely used in different applications [85]. In the k-NN classifier, an unknown sample is classified based on the similarity to the known, trained, or labeled samples by computing the distances between the unknown sample and all labeled samples. k-nearest samples are then selected as the basis for classification; and the unknown sample is assigned to the class which has the most samples among the k-nearest samples. For that, the k-NN classifier algorithm depends on: (a) integer k (number of neighbors)—changing the values of k parameter may change the classification result; (b) a set of labeled training data—thus, adding or removing any samples to the training samples will affect the final decision of k-NN classifier; and (c) a distance metric. In k-NN, the Euclidean distance is often used as the distance metric to measure the distance between two samples. k-NN classifier is analytically traceable and simple to implement, but one of the main problems of the k-NN algorithm is that it needs all the training samples to be in memory at run-time; for this reason, it is called memory-based classification [85,86].

The set of 240 spectra was randomly divided into two subsets: learning subset (180 cases) and validation subset (60 cases). The k-NN model is based on the spectral windows W1–W5. Calculations were performed using Statistica 12. The quality of the model was evaluated on the basis of quality indicators presented in Table 1.

Selected k-NN were validated in terms of: false negative (FN), true positive (TP), informedness, markedness, condition positive (P)—number of real positive cases in the data, condition negative (N)—number of real negative cases in the data. Also,

- sensitivity as well as true positive rate (TPR): TTR = $\frac{\mathrm{TP}}{P}$ = $\frac{\mathrm{TP}}{\mathrm{TP}+\mathrm{FN}}$ = 1 − FNR
- specificity as well as true negative rate (TNR): TNR = $\frac{\mathrm{TN}}{N}$ = $\frac{\mathrm{TN}}{\mathrm{TN}+\mathrm{FP}}$ = 1 − FPR
- miss rate as well as false negative rate (FNR): FNR = $\frac{\mathrm{FN}}{P}$ = $\frac{\mathrm{FN}}{\mathrm{FN}+\mathrm{TP}}$ = 1 − TPR
- false positive rate (FPR): FPR = $\frac{\mathrm{FP}}{N}$ = $\frac{\mathrm{FP}}{\mathrm{FP}+\mathrm{TN}}$= 1 − TNR
  where FP is a number of false positives, TN is the number of true negatives and N = FP + TN
- precision (PPV): PPV = $\frac{\mathrm{TP}}{\mathrm{TP}+\mathrm{FP}}$
- false omission rate (FOR): FOR = $\frac{\mathrm{FN}}{\mathrm{FN}+\mathrm{TN}}$ = 1 − NPV
- negative predictive value (NPV): NPV = $\frac{\mathrm{TN}}{\mathrm{TN}+\mathrm{FN}}$ = 1 − FOR
- false discovery rate (FDR): FDR = $\frac{\mathrm{FP}}{\mathrm{FP}+\mathrm{TP}}$ = 1 − PPV
- positive likelihood ratio (PLR): PLR = $\frac{\mathrm{TPR}}{100-\mathrm{TNR}}$
- negative likelihood ratio (NLR): NLR = $\frac{100-\mathrm{TPR}}{\mathrm{TNR}}$
- accuracy: accuracy = $\frac{\mathrm{TN}+\mathrm{TP}}{\mathrm{TN}+\mathrm{FP}+\mathrm{TP}+\mathrm{FN}}$

### 4.6. Hierarchical Cluster Analysis (HCA)

Hierarchical cluster analysis can be conceptualized as being agglomerative or divisive. Agglomerative hierarchical clustering separates each case into its own individual cluster in the first step so that the initial number of clusters equals the total number of cases [87]. At successive steps, similar cases—or clusters—are merged together until every case is grouped into one single cluster. Divisive hierarchical clustering works in the reverse manner with every case starting in one large cluster and gradually being separated into groups of clusters until each case is in an individual cluster [87].

HCA, an unsupervised method consisting of grouping the spectra with the same degree of similarity using the software Statistica 12, was also conducted to differentiate the serum spectra for noninfected or *H. pylori* infected animals. The chi-square statistical test was used to check the part of the IR spectra which correlated with the examined feature. Best predictors for *H. pylori* infection were used in HCA. Manhattan metric and Euclidean distances (inter-spectral distance) between all data were calculated and Ward’s algorithm was used to construct the dendrogram displaying the grouping of spectra into clusters based on heterogeneity values. Euclidean distances allow for the distance between two cases to be calculated across all variables and reflected in a single distance value. At each step in the procedure, the squared Euclidean distance between all pairs of cases and clusters is calculated and shown in a proximity matrix. At each step, the pair of cases or clusters with the smallest squared Euclidean distance will be joined with one another. Ward’s minimum variance method calculates the distance between cluster members and the centroid. The centroid of a cluster is defined as the point at which the sum of squared Euclidean distances between the point itself and each other point in the cluster is minimized [88].

## 5. Conclusions

Based on the chi-square test, 10 wavenumbers of IR spectra correlating with *H. pylori* infection were selected for HCA analysis by the use of the k-NN model. The sensitivity and specificity of this model were 97% and 100%, respectively, whereas the accuracy reached 98%. This study shows that the combination of infrared spectroscopy and HCA methods may potentially be useful for the differentiation of serum samples from guinea pigs uninfected or infected with *H. pylori* on the basis of TNF, CRP, transferrin, glucose, α2-globulins, and IgM, IgG1/4. Further study on a larger number of samples will allow standardization of this method for the analysis of guinea pig sera regarding *H. pylori* exposure in conjunction with the development of inflammatory response and antibody production as well as some metabolic markers such as glucose. The results obtained from the experimental model of *H. pylori* infection in guinea pigs will be helpful in adjusting this methodology to study the systemic effects of *H. pylori* local gastric infection in humans on the basis of different levels of selected soluble determinants in serum samples.

## Figures and Tables

**Table 1:** Selected examples of the use of attenuated total reflectance-infrared spectroscopy (ATR-IR) in biological samples analysis.

Lp	Aim of Research	Application	Organisms	Tested Biological Sample	References
1	Detection of endometrial cancer	endometrial cancer	Human	serum	[22]
2	Diagnosis of ovarian cancer	ovarian cancer	Human	serum	[23,24,25]
3	Diagnosis of breast cancer	breast cancer	Human	serum	[26]
4	Examination of leukemia patients	leukemia	Human	serum	[27]
5	Diagnosis of type 2 diabetes	type 2 diabetes	Human	serum	[28]
6	Differentiation of rheumatoid arthritis (RA) patients from healthy individuals	rheumatoid arthritis	Human	serum	[29]
7	Diagnosis and monitoring therapy of depression; Differences between dried and liquid blood serum samples	depression	Rat Human	serum	[30] [31]
8	Diagnosis of idiopathic Parkinson’s disease	idiopathic Parkinson’s disease	Human	serum	[32]
9	Detected biological marker Alzheimer’s disease	Alzheimer’s disease	Human	serum	[33,34]
10	Investigation of quantitative changes of selected soluble biomarkers, correlated with *H. pylori* infection in children and presumable consequent delayed growth	delayed growth	Human	serum	[35]
11	Differentiation of serum samples of opioid users from healthy individuals	opioid-driven disorders	Human	serum	[36]
12	Prognosis in patients with ascites and cirrhosis	ascites, cirrhosis	Human	serum	[37]
13	Qualitative and quantitative changes in phospholipids and proteins in olfactory bulbectomy	olfactory bulbectomy	Rat	serum	[38]
14	Biochemical analysis of acute lead poisoning	acute lead poisoning	Rat	serum	[39]
15	Analysis of serum immunoglobulins	analysis of immunoglobulins	Human	serum	[40]
16	Quantification of protein concentration	protein concentration	Human	serum	[41]
17	Differentiation of lung carcinoma (A549) cell line; Analysis of primary (Oral Squamous Carcinoma Cells, grade 3) OSCC_G3 cell line	in vitro drug activity	Human	cell line	[42,43] [44,45]
18	Differentiation of granulosa cells	ovarian endometriosis; oocytes	Human	cell line	[46] [47]
19	Identification of breast cancer and melanoma	breast cancer; melanoma	Human	cell line	[48]
20	Diagnostic of human pancreatic cancer	pancreatic cancer	Human	tissue	[49]
21	Diagnostic of neoplastic thyroid tissue	thyroid tissue	Human	tissue	[50]
22	Differentiation of urinary bladder cancer	urinary bladder cancer	Human	tissue	[51]

**Table 2:** The level of anti-*H. pylori* GE IgM and IgG (U/mL) in guinea pig sera.

Study Groups	*n*	*H. pylori* Negative	*H. pylori* Positive	* *p*-Value
anti-*H. pylori* IgM	60	20 (OD = 0.254 ± 0.061)	40 (OD = 0.610 ± 0.055)	0.0001
anti-*H. pylori* IgG	60	20 (OD = 0.462 ± 0.053)	40 (OD = 1.241 ± 0.051)	0.0001

Shown are mean values of optical density (OD) in enzyme linked immunosorbent assay (ELISA) ± standard deviation (SD). The differences between tested variables were assessed using Statistica 12 PL software with a nonparametric Mann–Whitney U test. The results were considered statistically significant when * *p* < 0.05.

**Table 3:** Molecules identified in the IR spectra of guinea pig sera.

Molecule	Selected Absorption Band [cm^−1^]	ID	Possible Chemical Bond	One of Possible Chemical Bond	Reference
Glucose	1062–997	[B1]	C-O symmetric stretching of glucose region C–O stretching	Carboxylic acids	[58]
A2 globulins	1060–1116	[B2]	C–C–C bending C–O stretching C–N stretching	Amino acid	[58]
IgM	1428–1360	[B3]	N–O symmetric stretching O–H bending methyl symmetric deformation	Carboxylic acid Amino acid	[59]
IgG1	1419–1361	[B4]	C–H rocking, C–C stretching methyl symmetric deformations	Hydrocarbons Amino acid	[59]
Transferrin	1428–1363	[B5]	CH_2_ wagging O–H bending	Carboxylic acid	[59]
IgG4	1538–1505	[B6]	CO_2_ asymmetric stretching C–N stretching, NH bending	Amide II	[59]
CRP	1541–1600	[B7]	C–C stretching NH bending N–H in plane bending vibration coupled to C–N stretching vibration protein	Amide II	[60]
TNF	1690–1636	[B8]	C=O symmetric stretching C=C stretching NH_2_ scissoring	Amide I	[61]

ID of the infrared spectra regions that are correlated with *H. pylori* infection in guinea pigs. B: band. Ig: immunoglobulin. CRP: C-reactive protein. TNF: tumor necrosis factor.

**Table 4:** Best predictors for *H. pylori* infection.

Window	Absorpcion Band [cm^−1^]	χ^2^-Square Test Value	*p*-Value (× 10^−5^)	One of Possible Chemical Bond
W4	1061	7.21	21.4	N–H bending
	1105	4.08	33.5	C–O stretching
W3	1394	15.22	21.1	asymmetric C–H, scissoring of –CH3
	1395	17.97	31.1	asymmetric C–H, scissoring of –CH3
	1400	18.51	20.9	O–H bending
	1412	15.21	17.7	CO_2_ asymmetric stretching
	1420	13.36	21.1	CO_2_ asymmetric stretching
W2	1522	16.30	14.4	C=C bending
	1541	18.31	23.2	C=C bending
	1630	14.3	25.5	NH_2_ scissoring

Satisfactory results for analysis of the IR spectra were obtained using the k-NNs algorithm. Proposed models are characterized by 96% accuracy. The summary results for the k-NN model are presented in Table 4.

**Table 5:** The results of the k-NN model for differentiation of *H. pylori* infected animals.

Model Details	
Number of nearest neighbors	1
Distance	Manhattan
Standardization	No
Averaging	Homogeneous
**Quality of the k-NN Model**	
Total number of spectra validation group	60
True positive (TP)	40
False Positive (FP)	0
False Negative (FN)	1
True negative (TN)	20
Sensivity	0.97
Miss rate	0.08
Specificity	1.00
Fall out	0.00
Precision	1.00
False discovery rate	0.00
False omission rate	0.05
Negative predictive value	0.92
Positive likelihood ratio	ND
Negative likelihood ratio	0.11
Diagnostic odds ratio	ND
Accuracy	0.98
Prevalence	0.55

## Data Availability

The data that support the findings of this study are available from the corresponding author upon reasonable request.

## Abbreviations

CagA	Cytotoxin-associated gene A
CCUG	Culture Collection University of Gothenburg
CPR	C-reactive protein
ELISA	Enzyme-linked immunosorbent assay
GE	Glycine acid extract
HCA	Hierarchical cluster analysis
HLO	*Helicobacter* like organism
IL	Interleukin
IR	Infrared spectroscopy
K-NN	K-nearest neighbors
LPS	Lipopolysaccharide
MALT	Mucosa-associated lymphoid tissue
NIR	near-infrared spectroscopy
PCR	Polymerase chain reaction
RUT	rapid urease test
TNF	Tumor necrosis factor
W	Wavenumber windows
VacA	Vacuolating cytotoxin A

## References

[1] Marshall B.J., Warren J.R. (1984). Unidentified curved bacilli in the stomach of patients with gastritis and peptic ulceration. Lancet.

[2] McColl K.E. (2010). Clinical practice. *Helicobacter pylori* infection. N. Engl. J. Med..

[3] Blaser M.J., Atherton J.C. (2004). *Helicobacter* infection persistence: Biology and disease. J. Clin. Investig..

[4] Peek R.M., Crabtree J.M. (2006). *H. pylori* infection and gastric neoplasia. J. Pathol..

[5] Posselt G., Backert S., Wessler S. (2013). The functional interplay of *H. pylori* factors with gastric epithelial cells induces a multi-step process in pathogenesis. Cell Commun. Signal.

[6] Suzuki N., Murata-Kamiya N., Yanagiya K., Suda W., Hattori M., Kanda H., Bingo A., Fujii Y., Maeda S., Koike K. (2015). Mutual reinforcement of inflammation and carcinogenesis by the *H. pylori* CagA oncoprotein. Sci. Rep..

[7] Chmiela M., Karwowska Z., Gonciarz W., Allushi B., Stączek P. (2017). Host pathogen interactions in *Helicobacter pylori* related gastric cancer. World J. Gastroenterol..

[8] Chmiela M., Michetti P. (2006). Inflammation, immunity, vaccines for *Helicobacter pylori* infection. Helicobacter.

[9] Michalkiewicz J., Helmin-Basa A., Grzywa R., Czerwionka-Szaflarska M., Szaflarska-Poplawska A., Mierzwa G., Marszalek A., Bodnar M., Nowak M., Dzierzanowska-Fangrat K. (2015). Innate immunity components and cytokines in gastric mucosa in children with *Helicobacter pylori* infection. Med. Inflamm..

[10] Grebowska A., Moran A.P., Bielanski W., Matusiak A., Rechcinski T., Rudnicka K., Baranowska A., Rudnicka W., Chmiela M. (2010). *Helicobacter pylori* lipopolysaccharide activity in human peripheral blood mononuclear leukocyte cultures. J. Physiol. Pharmacol..

[11] Chmiela M., Miszczyk E., Rudnicka K. (2014). Structural modifications of *Helicobacter pylori* lipopolysaccharide: An idea for how to live in peace. World J. Gastroenterol..

[12] Rudnicka K., Miszczyk E., Matusiak A., Walencka M., Moran A.P., Rudnicka W., Chmiela M. (2015). *Helicobacter pylori*-driven modulation of NK cell expansion, intracellular cytokine expression and cytotoxic activity. Innate Immun..

[13] Varbanova M., Frauensclager K., Malfertheiner P. (2014). Chronic gastritis—An update. Best Pract. Res. Clin. Gastroenterol..

[14] O’ Rourke J.L., Lee A. (2003). Animal models of *Helicobacter pylori* infection and disease. Microbes Infect..

[15] Peek R.M. (2008). *Helicobacter pylori* infection and disease: From humans to animal models. Dis. Models Mech..

[16] Bitter-Suermann D., Hoffman T., Burger R., Hadding U. (1981). Linkage of total deficiency of the second component (C2) of the complement system and of genetic C2 polymorphism to the major histocompatibility complex of the guinea pig. J. Immunol..

[17] D’Erchia A.M., Gissi C., Pesole G., Saccone C., Arnason U. (1996). The guinea pig is not a rodent. Nature.

[18] Wicher V., Scarroza A.M., Ramsing A.L., Wicher K. (1998). Cytokine gene expression in skin of susceptible guinea pigs infected with *Treponema pallidum*. Immunology.

[19] Whary M.T., Fox J.G. (2004). Natural and experimental *Helicobacter* infection. Comp. Med. Sci..

[20] Mantsch H.H., Chapman D. (1996). Infrared Ssectroscopy of Bbomolecules.

[21] Zhou Y.P., Xu L., Tang L.J., Jiang J.H., Shen G.L., Yu R.Q., Ozaki Y. (2007). Gas Chromatography-Inductively Coupled Plasma-Mass Spectrometry for Mercury Speciation in sea food. Anal. Sci..

[22] Paraskevaidi M., Morais C.L., Ashton K.M., Stringfellow H.F., McVey R.J., Ryan N.A., O’Flynn H., Sivalingam V.N., Kitson S.J., MacKintosh M.L. (2020). Detecting Endometrial Cancer by Blood Spectroscopy: A Diagnostic Cross-Sectional Study. Cancers.

[23] Owens G.L., Gajjar K., Trevisan J., Fogarty S.W., Taylor S.E., Da Gama-Rose B., Martin-Hirsch P.L., Martin F.L.J. (2014). Vibrational biospectroscopy coupled with multivariate analysis extracts potentially diagnostic features in blood plasma/serum of ovarian cancer patients. Biophotonics.

[24] Lima K.M., Gajjar K.B., Martin-Hirsch P.L., Martin F.L. (2015). Segregation of ovarian cancer stage exploiting spectral biomarkers derived from blood plasma or serum analysis: ATR-FTIR spectroscopy coupled with variable selection methods. Biotechnol. Prog..

[25] Gajjar K., Trevisan J., Owens G., Keating P.J., Wood N.J., Stringfellow H.F., Martin-Hirsch P.L., Martin F.L. (2013). Fourier-transform infrared spectroscopy coupled with a classification machine for the analysis of blood plasma or serum: A novel diagnostic approach for ovarian cancer. Analyst.

[26] Backhaus J., Mueller R., Formanski N., Szlama N., Meerpohl H.-G., Eidt M., Bugert P. (2010). Diagnosis of breast cancer with infrared spectroscopy from serum samples. Vib. Spectrosc..

[27] Erukhimovitch V., Talyshinsky M., Souprun Y., Huleihel M. (2006). FTIR spectroscopy examination of leukemia patients plasma. Vib. Spectros..

[28] Guang P., Huang W., Guo L., Yang X., Huang F., Yang M., Wen W., Li L. (2020). Blood-based FTIR-ATR spectroscopy coupled with extreme gradient boosting for the diagnosis of type 2 diabetes: A STARD compliant diagnosis research. Medicine.

[29] Lechowicz L., Chrapek M., Gaweda J., Urbaniak M., Konieczna I. (2016). Use of Fourier-transform infrared spectroscopy in the diagnosis of rheumatoid arthritis: A pilot study. Mol. Biol. Rep..

[30] Depciuch J., Sowa-Kucma M., Nowak G., Papp M., Gruca P., Misztak P., Parlinska-Wojtan M. (2017). Qualitative and quantitative changes in phospholipids and proteins investigated by spectroscopic techniques in animal depression model. Spectrochim. Acta Part A Mol. Biomol. Spectrosc..

[31] Depciuch J., Parlinska-Wojtan M. (2018). Comparing dried and liquid blood serum samples of depressed patients: An analysis by Raman and infrared spectroscopy methods. J. Pharm. Biomed. Anal..

[32] Schipper H.M., Kwok C.S., Rosendahl S.M., Bandilla D., Maes O., Melmed C., Rabinovitch D., Burns D.H. (2008). Spectroscopy of human plasma for diagnosis of idiopathic Parkinson’s disease. Biomark. Med..

[33] Schneider P., Hampel H., Buerger K. (2009). Biological marker candidates of Alzheimer’s disease in blood, plasma, and serum. Cns Neurosci. Ther..

[34] Peuchant E., Richard-Harston S., Bourdel-Marchasson I., Dartigues J.-F., Letenneur L., Barberger-Gateau P., ArnaudDabernat S., Daniel J.-Y. (2008). Infrared spectroscopy: A reagent-free method to distinguish Alzheimer’s disease patients from normal-aging subjects. Transl. Res..

[35] Gonciarz W., Lechowicz Ł., Urbaniak M., Kaca W., Chmiela M. (2020). Attenuated Total Reflectance Fourier Transform Infrared Spectroscopy (FTIR) and Artificial Neural Networks Applied to Investigate Quantitative Changes of Selected Soluble Biomarkers, Correlated with *H. pylori* Infection in Children and Presumable Consequent Delayed Growth. J. Clin. Med..

[36] Guleken Z., Ünübol B., Bilici R., Sarıbal D., Toraman S., Gündüz O., Kuruca S.E. (2020). Investigation of the discrimination and characterization of blood serum structure in patients with opioid use disorder using IR spectroscopy and PCA-LDA analysis. J. Pharm. Biomed. Anal..

[37] Le Corvec M., Jezequel C., Monbet V., Fatih N., Charpentier F., Tariel H., Boussard-Plédel C., Bureau B., Loréal O., Sire O. (2017). Mid-infrared spectroscopy of serum, a promising non-invasive method to assess prognosis in patients with ascites and cirrhosis. PLoS ONE.

[38] Depciuch J., Parlinska-Wojtan M. (2018). Qualitative and quantitative changes in phospholipids and proteins investigated by spectroscopic techniques in olfactory bulbectomy animal depression model. J. Pharm. Biomed. Anal..

[39] Tian W., Wang D., Fan H., Yang L., Ma G. (2008). A Plasma Biochemical Analysis of Acute Lead Poisoning in a Rat Model by Chemometrics-Based Fourier Transform Infrared Spectroscopy: An Exploratory Study. Front. Chem..

[40] Sankari G., Krishnamoorthy E., Jayakumaran S., Gunasekaran G., Priya V.V., Subramaniam S., Subramaniam S., Mohan S.K. (2010). Analysis of serum immunoglobulins using Fourier transform infrared spectral measurements. Res. Art. Biol. Med..

[41] Spalding K., Bonnier F., Bruno C., Blasco H., Board R., Benz-de Bretagne H.I., Byrnef H., Butlera J., Chourpab I., Radhakrishnan R. (2008). Enabling quantification of protein concentration in human serum biopsies using attenuated total reflectance–Fourier transform infrared (ATR-FTIR) spectroscopy. Vib. Spectrosc..

[42] Byrne H.J., Bonnier F., Casey A., Maher M., McIntyre J., Efeoglu E., Farhane Z. (2008). Advancing Raman microspectroscopy for cellular and subcellular analysis: Towards in vitro high-content spectralomic analysis. Appl. Opt..

[43] Efeoglu E., Maher M.A., Casey A., Hugh J., Byrne H.J. (2018). Toxicological assessment of nanomaterials: The role of in vitro Raman microspectroscopic analysis. Anal. Bioanal. Chem..

[44] Giorgini E., Sabbatini S., Rocchetti R., Notarstefano V., Rubini C., Conti C., Orilisi G., Mitri E., Bedolla D.E., Vaccari L. (2018). In vitro FTIR microspectroscopy analysis of primary oral squamous carcinoma cells treated with cisplatin and 5-fluorouracil: A new spectroscopic approach for studying the drug–cell interaction. Analyst.

[45] Notarstefano V., Sabbatini S., Pro C., Belloni A., Orilisi G., Rubini C., Byrne H.J., Vaccari L., Giorgini E. (2020). Exploiting fourier transform infrared and Raman microspectroscopies on cancer stem cells from oral squamous cells carcinoma: New evidence of acquired cisplatin chemoresistance. Analyst.

[46] Notarstefano V., Gioacchini G., Byrne H.J., Zacà C., Sereni E., Vaccari L., Borini C., Carnevali O., Giorgini E. (2019). Vibrational characterization of granulosa cells from patients affected by unilateral ovarian endometriosis: New insights from infrared and raman microspectroscopy. Spectrochim. Acta Part A Mol. Biomol. Spectrosc..

[47] Gioacchini G., Notarstefano V., Sereni E., Zacà C., Coticchio G., Giorgini E., Vaccari L., Carnevali O., Borini A. (2018). Does the molecular and metabolic profile of human granulosa cells correlate with oocyte fate? New insights by Fourier transform infrared microspectroscopy analysis. Mol. Hum. Reprod..

[48] Verdonck M., Wald N., Janssis J., Yan P., Meyer C., Legat A., Speiser D.E., Desmedt C., Larsimont D., Sotoriou C. (2013). Breast cancer and melanoma cell line identification by FTIR imaging after formalin-fixation and paraffin-embedding. Analyst.

[49] Notarstefano V., Sabbatini S., Conti C., Pisani M., Astolfi P., Pro C., Rubini C., Vaccari L., Giorgini E. (2020). Investigation of human pancreatic cancer tissues by Fourier Transform Infrared Hyperspectral Imaging. J. Biophotonics.

[50] Depciuch J., Stanek-Widera A., Lange D., Biskup-Frużyńska M., Stanek-Tarkowska J., Czarny W., Cebulski J. (2018). Spectroscopic analysis of normal and neoplastic (WI-FTC) thyroid tissue. Spectrochim. Acta Part A Mol. Biomol. Spectrosc..

[51] Hughes C., Iqbal-Wahid J., Brown M., Shanks J.H., Eustace A., Denley H., Hoskin P.J., West C., Noel W., Gardner P. (2013). FTIR microspectroscopy of selected rare diverse sub-variants of carcinoma of the urinary bladder. J. Biophotonics.

[52] Naumann D., Helm D., Labischinski H., Giesbrecht P., Nelson W. (1991). The characterization of microorganisms by Fourier-transform infrared spectroscopy (FT-IR) In Modern Techniques for Rapid Microbiological Analysis.

[53] Cooper E.A., Knutson K. (1995). Fourier transform infrared spectroscopy investigations of protein structure. Pharm. Biotechnol..

[54] Naumann D., Meyers R. (2000). Infrared Spectroscopy in Microbiology. Encyclopedia of Analytical Chemistry.

[55] Banyay M., Sarkar M., Graslund A. (2003). A library of IR bands of nucleic acids in solution. Biophys. Chem..

[56] Gonciarz W., Krupa A., Hinc K., Obuchowski M., Moran A.P., Gajewski A., Chmiela M. (2019). The effect of *Helicobacter pylori* infection and different *H. pylori* components on the proliferation and apoptosis of gastric epithelial cells and fibroblasts. PLoS ONE.

[57] Gonciarz W., Krupa A., Chmiela M. (2020). Proregenerative Activity of IL-33 in Gastric Tissue Cells Undergoing *Helicobacter Pylori*-Induced Apoptosis. Int. J. Mol. Sci..

[58] Deleris G., Petibois C. (2003). Applications of FT-IR spectrometry to plasma contents analysis and monitoring. Vib. Spectrosc..

[59] Petibois C., Cazorla G., Cassaigne A., Déléris G. (2001). Plasma protein contents determined by Fourier-transform infrared spectrometry. Clin. Chem..

[60] Anderson O., Viberg P., Forsberg P., Nikolajeff F., Osterlund L., Klarsson M. (2006). Nanocrystalline diamond sensor targeted for selective CRP detection: An ATR-FTIR spectroscopy study. Anal. Bioanal. Chem..

[61] Reiter G., Hassler N., Weber V., Falkenhagen D., Fringeli U.P. (2004). In situ FTIR ATR spectroscopic study of the interaction of immobilized human tumor necrosis factor-α with a monoclonal antibody in aqueous environment. Biochim. Biophys. Acta Proteins Proteom..

[62] Bielanski W., Konturek S.J. (1996). New approach to 13C urea breath test capsule-based modification with low dose of ^13^C urea in the diagnosis of *Helicobacter pylori* infection. J. Physil. Pharmacol..

[63] Wisniewska M., Nilsson H.O., Bak-Romaniszyn L., Rechcinski T., Bielanski W., Planeta-Malecka I., Plonka M., Konturek S., Wadstrom T., Rudnicka W. (2001). Detection of specific *Helicobacter pylori* DNA and antigens in stool samples in dyspeptic patients and health subjects. Microbiol. Immunol..

[64] Malfertheiner P., Megraud F., O’Morain C.A., Atherton J., Axon A.T.R., Bazzoli F., Gensini G.F., Gisbert J.P., Graham D.Y., Rokkas T. (2012). The European Helicobacter Study Group (EHSG). Management of *Helicobacter pylori* infection-the Maastricht IV/Florence Consensus Report. Gut.

[65] Chisholm S.A., Owen R.J., Teaure E.L., Saverymuttu S. (2001). PCR-based diagnosis of *Helicobacter pylori* infection and real-time determinantion of claritromycin resistance directly from human gastric biopsy samples. J. Clin. Microbiol..

[66] Di Bonaventura G., Piccolomini R., Pompilio A., Zappacosta R., Picolomini M., Neri M. (2007). Serum and mucosal cytokine profiles in patients with active *Helicobacter pylori* and in ischemic heart disease: Is there a relationship?. Int. J. Immunopathol. Pharmacol..

[67] Bravo D., Hoare A., Soto C., Valenzuela M.A., Quest A.F.G. (2018). *Helicobacter pylori* in human health and diseases: Mechanisms for local gastric and systemic effects. World J. Gastroenterol..

[68] Ghimire H., Venkataramani M., Bian Z., Liu Y., Perera A.U. (2017). ATR-FTIR spectral discrimination between normal and tumorous mouse models of lymphoma and melanoma from serum samples. Sci. Rep..

[69] Lemes L.C., Caetano Júnior P.C., Strixino J.F., Aguiar J., Raniero L. (2016). Analysis of serum cortisol levels by Fourier Transform Infrared Spectroscopy for diagnosis of stress in athletes. Res. Biomed. Eng..

[70] Zhou J., Wang Z., Sun S., Liu M., Zhang H. (2001). A rapid method for detecting conformational changes during differentiation and apoptosis of HL60 cells by Fourier transform infrared spectroscopy. Biotechnol. Appl. Biochem..

[71] Shen Y.C., Davies A.G., Linfield E.H., Elsey T.S., Taday P.F., Arnone D.D. (2003). The use of Fourier-transform infrared spectroscopy for the quantitative determination of glucose concentration in whole blood. Phys. Med. Biol..

[72] Miszczyk E., Walencka M., Chmiela M. (2011). Modele zwierzęce w badaniach nad przebiegiem zakażeń *Helicobacter pylori*. Post. Hig. Med. Dośw..

[73] Miszczyk E., Walencka M., Rudnicka K., Matusiak A., Rudnicka W., Chmiela M. (2014). Antigen-specific lymphocyte as a marker of immune response in guinea pigs with sustained *Helicobacter pylori* infection. Acta Biochim. Pol..

[74] Jafarzadeh A., Hassanshahi G.H., Nemati M. (2009). Serum levels of high-sensitivity C-reactive protein (hs-CRP) in *Helicobacter pylori*-infected peptic ulcer patients and its association with bacterial CagA virulence factor. Dig. Dis. Sci..

[75] Ishida Y., Suzuki K., Taki K., Niwa T., Kurotsuchi S., Ando H., Hamajima N. (2008). Significant association between *Helicobacter pylori* infection and serum C-reactive protein. Int. J. Med. Sci..

[76] Leontiadis G.I., Sharma V.K., Howden C.W. (1999). Non–Gastrointestinal Tract Associations of *Helicobacter pylori* Infection: What Is the Evidence?. Arch. Intern. Med..

[77] Shibata J., Goto H., Arisawa T., Niwa Y., Hayakawa T., Nakayama A., Mori N. (1999). Regulation of tumor necrosis factor (TNF) induced apoptosis by soluble TNF receptor in *Helicobacter pylori* infection. Gut.

[78] Ko G.T., Chan F.K., Chan W.B., Sung J.J., Tsoi C.L., To K.F., Lai C.W., Cockram C.S. (2001). *Helicobacter pylori* infection in Chinese subjects with type 2 diabetes. Endocr. Res..

[79] Peach H.G., Barnett N.E. (2001). *Helicobacter pylori* infection and fasting plasma glucose concentration. J. Clin. Pathol..

[80] Devrajani B.R., Shah S.Z., Soomro A.A., Devrajani T. (2010). Type 2 diabetes mellitus: A risk factor for *Helicobacter pylori* infection: A hospital based case-control study. Int. J. Diabetes. Dev. Ctries.

[81] Cardenas V.M., Mulla Z.D., Ortiz M., Graham D.Y. (2006). Iron deficiency and *Helicobacter pylori* infection in the United States. Am. J. Epidem..

[82] Qu X.H., Huang X.L., Xiong P., Zhu C.Y., Huang Y.L., Lu L.G., Lin H. (2010). Does *Helicobacter pylori* infection play a role in iron deficiency anemia? A meta-analysis. World J. Gastroenterol..

[83] Chmiela M., Ławnik M., Czkwianianc E., Rechciński T., Płaneta-Małecka I., Rudnicka W. (1998). Systemic humoral response to Helicobacter pylori in children and adults. Arch. Immunol. Ther. Exp..

[84] Rechcinski T., Chmiela M., Małecka-Panas E., Płaneta-Małecka I., Rudnicka W. (1997). Serological indicators of *Helicobacter pylori* infection in adult dyspeptic patients and health blood donors. Microbiol. Immunol..

[85] Duda R.O., Hart P.E., Stork D.G. (2012). Pattern Classification.

[86] Mitchell T.M. (1997). Machine Learning.

[87] Norusis M.J. (2010). Chapter 16: Cluster Analysis. PASW Statistics 18 Statistical Procedures Companion.

[88] Rencher A.C. (2002). Methods of Multivariate Analysis.

